# Clinical correlates and metabolic indicators of elevated fasting glucose in overweight/obese Chinese Han patients with first-episode and drug-naive major depressive disorder

**DOI:** 10.3389/fendo.2023.1102670

**Published:** 2023-03-15

**Authors:** Wenqi Gao, Zhifang Deng, Xiaonan Cai, Dan Zhang, Han Xiao, Xiangyang Zhang

**Affiliations:** ^1^Institute of Maternal and Child Health, Wuhan Children’s Hospital (Wuhan Maternal and Child Healthcare Hospital), Tongji Medical College, Huazhong University of Science and Technology, Wuhan, Hubei, China; ^2^Department of Pharmacy, The Central Hospital of Wuhan, Tongji Medical College, Huazhong University of Science and Technology, Wuhan, China; ^3^Woman Healthcare Department for Community, Wuhan Children’s Hospital (Wuhan Maternal and Child Healthcare Hospital), Tongji Medical College, Huazhong University of Science and Technology, Wuhan, Hubei, China; ^4^CAS Key Laboratory of Mental Health, Institute of Psychology, Beijing, China; ^5^Department of Psychology, University of Chinese Academy of Sciences, Beijing, China

**Keywords:** fasting glucose, overweight/obese, first-episode and drug-naïve major depressive disorder, Chinese Han, BMI

## Abstract

**Background:**

Overweight/obese major depressive disorder (MDD) patients have a high probability of developing glucose metabolism disorders; however, the results are inconsistent due to the confounding variables involved in the studies. The purpose of this study was to explore the prevalence and risk factors for elevated fasting glucose in Chinese Han patients with overweight/obese first-episode and drug naïve (FEDN) MDD.

**Methods:**

The study used a cross-sectional design and recruited 1718 FEDN MDD patients between the ages of 18 and 60 years. Socio-demographic information, anthropometric data, and biochemical parameters were collected. The 17-item Hamilton Assessment Scale for Depression (HAMD), the 14-item Hamilton Anxiety Scale (HAMA), and the Positive and Negative Syndrome Scale (PANSS) positive subscale were used to assess symptoms of all patients.

**Results:**

MDD patients with elevated fasting glucose had higher TSH, TPOAb, TC, TG, LDL-C, systolic and diastolic blood pressure levels than those with normal fasting glucose. Logistic regression analysis showed that age, TSH, TgAb, TPOA, and TG were related factors for elevated fasting glucose, while TSH and combination all these five parameters had the potential to differentiate between patients with elevated fasting glucose and those with normal fasting glucose. Multifactorial regression analysis showed that TSH, TG, and LDL-C were independently associated with elevated fasting glucose.

**Conclusion:**

Our findings suggest a high prevalence of elevated fasting glucose in overweight/obese FEDN MDD patients. Several clinically relevant factors and metabolic parameters are associated with elevated fasting glucose in overweight/obese FEDN MDD patients.

**Limitation:**

Due to the cross-sectional design, no causal relationship could be derived.

## Introduction

Major depressive disorder (MDD) is a severe neuropsychiatric disorder characterized by persistent low mood, lack of interest and loss of pleasure. The number of people suffering from MDD is increasing every year, and by 2030, depression will be the second largest burden of disease worldwide (1). Depression can negatively affect the patient’s clinical parameters, especially glucose and lipid metabolism indicators (2). Despite the widespread use of antidepressants, however, only 30% of patients achieve remission with initial antidepressant therapy (3). Therefore, there must be other unrevealed mechanisms and pathologies underlying depression that need to be explored.

The relationship between MDD and disorders of glucose metabolism has been widely explored, and as early as 1931, McCowan et al. observed that patients with MDD had concomitant abnormalities in glucose tolerance that improved with remission of depressive symptoms (4). The relative risk of developing diabetes in patients with MDD is 1.2-2.6 times higher than that of the healthy population (5). Research evidence suggests that depressive symptoms can trigger disturbances in glucose metabolism in individuals (6). Patients with MDD may have varying degrees of abnormal glucose metabolism, including elevated or reduced fasting glucose, insulin and glucagon levels (7, 8). On the other hand, patients with diabetes are twice as likely to develop MDD as normal healthy people (9). High levels of HbA1c and poor glycemic control are associated with an increased incidence of MDD (10, 11). The glucose metabolic status of patients with type 2 diabetes also improves or worsens with changes in the severity of depressive symptoms (12).

The correlation between depressive symptoms and glucose metabolism is related to a variety of factors, including signaling pathways, genetic factors, and especially metabolic factors. In the last 20 years, obesity/overweight is becoming one of the causes of health impairment and a major cause of glucose metabolism disorders (13). Furthermore, MDD patients have a 58% increased risk of developing obesity (14). In addition, obese or overweight MDD patients have a lower quality of life and a poorer prognosis (15). Evidence from our recent study showed that among Chinese Han FEDN MDD patients, obese or overweight individuals have a 55% increased risk of developing MDD compared to normal healthy individuals (14).

Several studies have reported on the relationship between overweight/obesity, MDD, and glucose metabolism disorders, but the results have been inconsistent. For example, Hryhorczuk et al. found that abnormal glucose metabolism resulting from central obesity may be the main reason for the increased incidence of MDD in obese patients (16). Haleem et al. reported that fasting glucose levels were higher in obese men with depression than in non-depressed patients (17). However, a German study with 4597 participants and a Dutch study with 4747 participants both reported no association between depressive symptoms and diabetes (18, 19). Aujla et al. found that the prevalence of depressive symptoms was not higher in the diabetic population than in the normal healthy population (20).

The variables involved in these studies that led to inconsistent results may be due to differences between the subjects included in the studies, such as patients being at different stages of the disease and being of different ethnicities. Also, the methods of blood biochemistry tests used and the criteria for normal groups differed between races, regions, and hospitals. More importantly, there are more risk factors that affect the relationship between obesity/overweight, MDD, and glucose metabolism disorders. mcmartin et al. reported an inverse relationship between fruit and vegetable intake and MDD, and that poor diet can negatively affect mental health disorders (21). Second, exercise may reduce depression by distracting from depressive symptoms and promoting self-efficacy. Dietary control and exercise also moderately lower postprandial glucose and insulin, and are more effective in people with higher BMI (22). slyepchenko et al. suggested that environmental triggers, such as excessive psychological stress, sleep disturbance, poor diet, physical inactivity, and smoking, as well as medical factors, including autoimmune diseases, contribute to a greater risk of obesity/overweight and glucose metabolism disorders in people with MDD (23). Therefore, we wanted to investigate whether overweight/obese patients with MDD are more likely to develop glucose metabolism abnormalities and what factors are involved in this pathogenesis.

First-episode and drug-naïve (FEDN) MDD patients may provide an exceptional opportunity to examine which risk factors are associated with glucose metabolism disorders occurring in overweight/obese MDD patients, while minimizing the impact of confounding factors, including disease duration, comorbidities, and medications. To our knowledge, no studies have been reported in the Chinese Han population about glucose metabolism disorders in overweight/obese FEDN MDD patients. Therefore, this is the first study to examine the incidence of elevated fasting glucose and clinically relevant factors in Chinese Han overweight/obese FEDN MDD patients.

It is hoped that the results of this study will help to identify biomarkers of glucose metabolism disorders in overweight/obese MDD patients. Therefore, it is hoped that the results of this study will help clinical staff to improve daily screening and early prevention to prevent patients from developing complications such as diabetes mellitus.

## Methods

### Participants

A cross-sectional design was used for this study. The ethics committee of the First Hospital of Shanxi Medical University reviewed and approved this study. All participants in this study were recruited from the Department of Psychiatry, First Hospital of Shanxi Medical University between 2015 and 2017. All participants signed a written informed consent form and had the right to decide whether to withdraw at any time if they wished.

Patients who met the following criteria were included in this study: (1) males and females between the ages of 18-60 years and of Chinese Han ethnicity; (2) diagnosis of MDD by two trained psychiatrists using the Structured Clinical Interview for DSM-IV (SCID); (3) had not received any prior medication treatment; (4) a score of ≥24 on the 17-item Hamilton Depression Scale (HAMD) and a disease duration of no more than 2 years; (5) no pregnant or breastfeeding female patients. First, MDD patients with BMI ≤ 16 kg/m^2^ or BMI≥35 kg/m^2^ were excluded. Second, patients were excluded if their hormone levels were below and above the normal group mean ± 3 standard deviations. Finally, in our present study, patients themselves reported whether they fasted or not, which was confirmed by their family members. Patients were not included in this study if they were not observed to be fasting.

**Figure 1:**
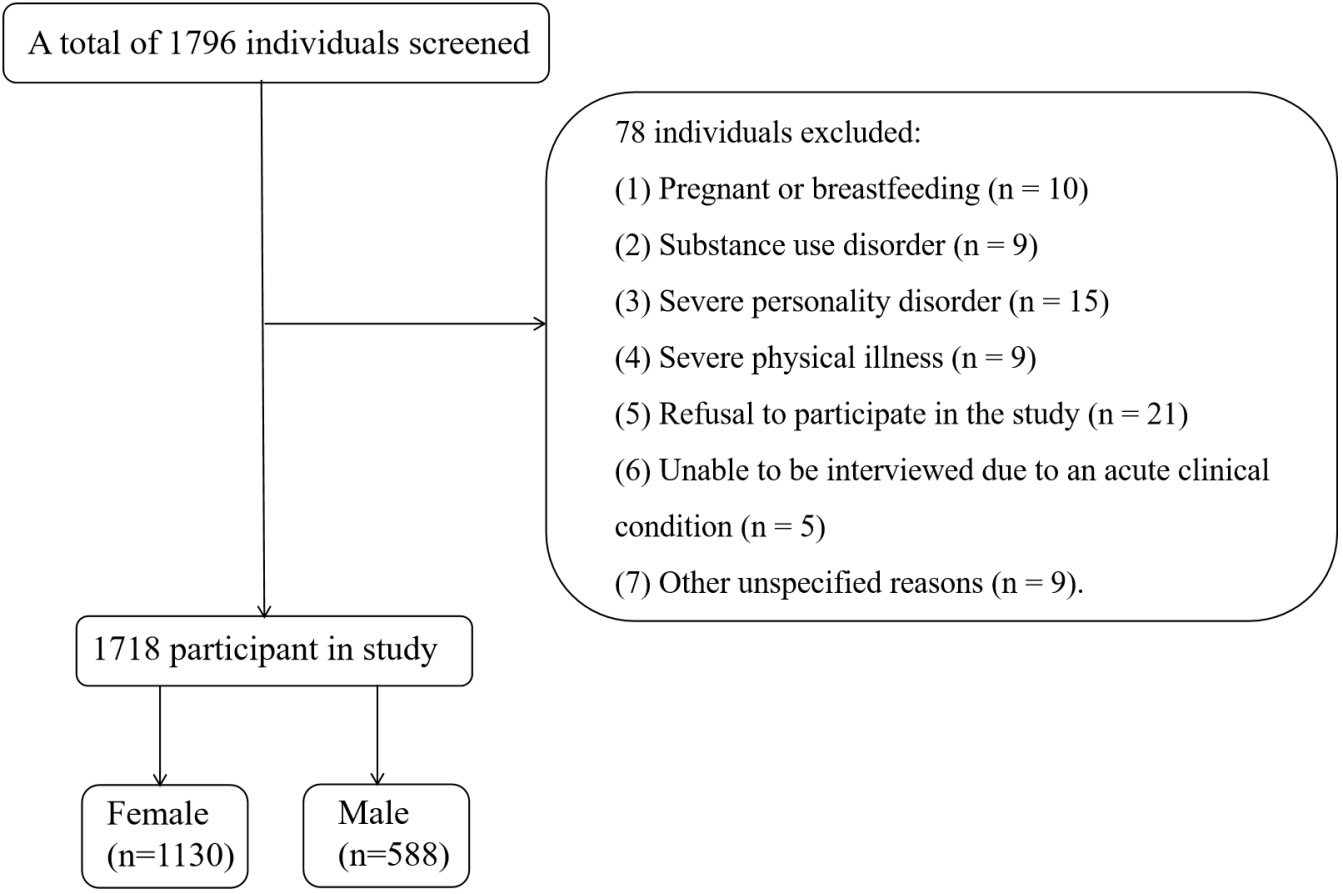
The flow chart to reflect the inclusion and discharge process.

Patients were excluded if they met any of the following criteria: (1) having a central nervous system disorder, neurodegenerative disease, or psychiatric disorder on Axis I diagnosis; (2) using immunosuppressive drugs; (3) having drug or alcohol abuse or dependence, as defined by self-reported substance use and medical records, but not smoking; and (4) refusing to sign a written consent form. Ultimately, a total of 1718 participants were included in this study. In Summary, we drew a flow chart to reflect the inclusion and discharge process more vividly (**Figure 1**).

### Clinical interview and assessment

Two independent psychiatrists were trained on how to use the PANSS, the HAMA, and the HAMD prior to the start of this study. After training, two psychiatrists’ inter-rater correlation coefficients for HAMD, HAMA, and PANSS positive subscale scores all exceeded 0.8. HAMD was used to assess depressive symptoms. In our study, a 24-point cut-off was used to classify participants as having (≥24 points) or not having depressive symptoms (<24 points) (24). The presence and severity of psychotic symptoms were assessed using the PANSS positive subscale score, with the total score ranging from 7 to 49. Patients with a total score of 15 or more were defined as having psychotic symptoms (25). In addition, a self-designed questionnaire was used to collect sociodemographic information, including gender, age, age of onset, duration of illness, marital status, and education level.

### Measurement of physical and biochemical parameters

Clinical data included body mass index (BMI), systolic blood pressure (SBP) and diastolic blood pressure (DBP). BMI was calculated by dividing body weight (in kilograms, with participants wearing light clothing) by the square of height (in centimeters, with participants standing upright and barefoot). All participants were divided into normal BMI group (BMI <24 kg/m^2^), overweight/obese group (24<=BMI <28 kg/m^2^ and BMI ≥28 kg/m^2^) according to the criteria developed by the Working Group on Obesity in China (WGOC) (26). Systolic and diastolic blood pressures in the left arm were recorded using an Omron Hem digital monitor while the patient was in a sitting position. The average of the two measurements was taken as the final blood pressure value of the individual.

Venous blood was collected from all participants between 6 am and 8 am after an overnight fast to measure biochemical parameters, including total cholesterol (TC), triglycerides (TG), high-density lipoprotein cholesterol (HDL-C), low-density lipoprotein cholesterol (LDL-C), fasting blood glucose (FBG), thyroid stimulating hormone (TSH), free triiodothyronine (FT3), free thyroxine (FT4), anti-thyroglobulin antibody (TgAb) and thyroid peroxidase antibody (TPOAb). Glucose oxidase and enzymatic colorimetric assays were used to detect fasting blood glucose levels and lipid profiles. Electrochemiluminescent immunoassay (Roche Diagnostics, Indianapolis, IN, USA) was used to determine serum concentrations of TSH, FT3, FT4, TPOAb and TgAb.

The normal ranges of the biochemical indicators were: fasting blood glucose: 3.89~6.11mmol/l; TSH: 0.27~4.20 mIU/L; FT3: 3.10~6.8 pmol/L; FT4: 10~23 pmol/L; TC: 2.81~6.17mmol/l; TG: 0~1.7mmol/l; HDL-C: 1.04~1.55mmol/l; LDL-C: 2.07~3.37 mmol/l.

### Statistical analysis

Analysis of categorical and continuous variables was performed using chi-square test and analysis of variance (ANOVA). The Kolmogorov-Smirnov one-sample test and Mann-Whitney U test were applied to normally and non-normally distributed variables. Bonferroni correction was used to adjust for multiple testing.

To explore risk factors for elevated fasting glucose in overweight/obese patients with FEDN MDD, univariate analyses were performed on normal and abnormal fasting glucose subgroups, followed by logistic regression analyses (Backward: Wald) for variables with significant differences. The area under receiver operating characteristic (AUCROC) was used to screen for significant variables and to determine whether the variable had the potential to distinguish between those with normal and abnormal fasting glucose. A consistency statistic between 0.7 and 0.8 is generally considered acceptable (27).

Finally, we performed multivariate regression analysis to detect associations between elevated fasting glucose and clinical and biochemical correlates in MDD patients. All statistical analyses were performed using SPSS version 23.0 (IBM, Chicago, IL, USA) and then graphs were plotted using GraphPad Prism 6.0. *P*-values were set to two-tailed with a significance level of α = 0.05.

## Results

### Prevalence of elevated fasting glucose in overweight/obese MDD patients and MDD patients with normal BMI

The percentage of overweight/obese MDD patients was 59.72% (1026/1718). Overweight/obese MDD patients had higher HAMA and HAMD scores, as well as positive subscale score compared to patients with normal BMI (all P<0.001). Fasting glucose was higher in overweight/obese MDD patients than in patients with normal BMI (P<0.001).

Among 1026 overweight/obese MDD patients, 153 patients (14.91%) had elevated fasting glucose levels. Among 692 MDD patients with normal BMI, 77 patients (11.13%) had elevated fasting glucose levels.

### Clinical characteristics and biochemical parameters of elevated fasting glucose in overweight/obese MDD patients

As shown in **Table 1**, univariate analysis showed significant differences between patients with normal fasting glucose and those with elevated fasting glucose in terms of demographic and clinical indicators, including age, age at onset, HAMA score, HAMD score, and PANSS positive symptom score (all P<0.001).

**Table 1:** Socio-demographics and clinical characteristics between overweight/obese MDD patients with elevated fasting glucose and normal fasting glucose.

Variables	Overweight/obese MDD patients		F/*X*^2^	p
	Elevated fasting glucose (*N* = 153)	Normal fasting glucose (*N* = 873)		
Age	37.58 ± 12.47	35.2 ± 12.09	5.02	0.03
Gender			0.12	0.73
male, n (%)	50 (32.68)	298 (34.14)		
female, n (%)	103 (67.32)	575 (65.86)		
Education			3.26	0.35
Junior high school, n (%)	46 (30.07)	212 (24.28)		
High school, n (%)	64 (41.83)	388 (44.44)		
University degree, n (%)	34 (22.22)	232 (26.58)		
Master’s degree, n (%)	9 (5.88)	41 (4.70)		
Marital status			1.48	0.22
Single, n (%)	35 (22.88)	241 (27.61)		
Married, n (%)	118 (11.12)	632 (72.39)		
Age of onset, years	37.39 ± 12.37	34.97 ± 11.97	5.27	0.02
Illness duration, months	6.42 ± 4.42	6.60 ± 4.93	0.18	0.67
HAMD	31.25 ± 2.64	30.21 ± 2.90	17.26	<0.001
HAMA	22.01 ± 3.83	20.64 ± 3.49	19.51	<0.001
Psychotic positive score	10.25 ± 5.67	8.74 ± 4.28	14.53	<0.001
TSH, μIU/L	7.26 ± 2.66	5.10 ± 2.19	118.13	<0.001
TgAb, IU/L	117.74 ± 285.23	85.74 ± 242.72	2.14	0.14
TPOAb, IU/L	141.41 ± 300.80	61.94 ± 125.29	30.68	<0.001
FT3, pmol/L	4.85 ± 0.70	4.93 ± 0.74	1.48	0.23
FT4, pmol/L	16.94 ± 3.04	16.74 ± 3.04	0.61	0.44
TC, mmol/L	5.811 ± 1.11	5.21 ± 1.07	41.10	<0.001
TG, mmol/L	2.48 ± 1.01	2.15 ± 0.93	16.51	<0.001
HDL-C, mmol/L	1.10 ± 0.29	1.23 ± 0.28	26.54	<0.001
LDL-C, mmol/L	3.34 ± 0.95	2.94 ± 0.85	27.53	<0.001
Systolic BP, mmHg	124.56 ± 9.74	119.98 ± 10.36	25.90	<0.001
Diastolic BP, mmHg	78.32 ± 6.84	76.06 ± 6.44	15.76	<0.001

Univariate analysis was used for statistical analysis. HAMD, Hamilton Rating Scale for Depression; HAMA, Hamilton Anxiety Rating Scale; TSH, thyroid stimulating hormone; TgAb, antithyroglobulin; TPOAb, thyroid peroxidases antibody; FT3, free triiodothyronine; FT4, free thyroxine; TC, total cholesterol; TG, triglycerides; HDL-C, high density lipoprotein cholesterol; LDL-C, low density lipoprotein cholesterol; BP, blood pressure.

Patients with elevated fasting glucose had higher TSH, TPOAb, TC, TG, LDL-C, systolic and diastolic blood pressure levels but lower HDL-C compared to patients with normal fasting glucose (all P<0.001, all P_bonferroni correction <_0.001).

Next, we compared the differences in demographic and clinical indicators between patients with normal fasting glucose and those with elevated fasting glucose grouped by sex and BMI quartiles, and the results are shown in **Tables 2**, **3**.

**Table 2:** BMI quartiles in socio-demographics and clinical characteristics between overweight/obese MDD patients with elevated fasting glucose and normal fasting glucose.

Variables	MDD with overweight (*N* = 1026)				BMI F/X^2^ (p)
	1 (*N* = 270)	2 (*N* = 248)	3 (*N* = 253)	4 (*N* = 255)	
Age	36.34 ± 12.15	35.40 ± 12.07	34.71 ± 12.02	34.27 ± 12.10	1.238 (0.29)
Age of onset, years	36.11 ± 12.02	35.12 ± 11.95	34.48 ± 11.88	34.09 ± 12.01	1.198 (0.31)
Illness duration, months	6.60 ± 4.99	6.94 ± 5.44	6.61 ± 4.67	6.28 ± 4.59	0.702 (0.55)
HAMD	30.06 ± 2.90	30.08 ± 3.02	30.22 ± 2.79	30.49 ± 2.90	0.517 (0.67)
HAMA	20.46 ± 3.48	20.50 ± 3.38	20.64 ± 3.41	20.95 ± 3.66	0.352 (0.79)
Psychotic positive score	8.29 ± 3.45	8.71 ± 4.37	8.93 ± 4.52	9.06 ± 4.72	0.363 (0.78)
Suicide attempts	0.10 ± 0.32	0.15 ± 0.43	0.17 ± 0.47	0.16 ± 0.42	0.532 (0.66)
CGI	5.86 ± 0.74	5.87 ± 0.76	5.94 ± 0.74	5.99 ± 0.75	0.596 (0.62)
TSH, μIU/L	4.81 ± 2.17	5.05 ± 2.18	5.15 ± 2.29	5.41 ± 2.08	0.456 (0.71)
TgAb, IU/L	99.64 ± 355.27	77.13 ± 190.99	89.20 ± 212.65	75.75 ± 151.40	0.094 (0.96)
TPOAb, IU/L	52.50 ± 106.18	46.62 ± 96.50	67.08 ± 138.82	81.36 ± 149.60	2.844 (0.04)
FT3, pmol/L	4.91 ± 0.73	4.92 ± 0.72	5.00 ± 0.77	4.89 ± 0.74	0.150 (0.93)
FT4, pmol/L	16.51 ± 2.99	16.64 ± 3.07	16.88 ± 2.94	16.93 ± 3.16	0.601 (0.61)
TC, mmol/L	5.17 ± 1.08	5.08 ± 1.10	5.25 ± 1.07	5.32 ± 1.02	0.643 (0.59)
HDL-C, mmol/L	1.23 ± 0.29	1.21 ± 0.27	1.24 ± 0.27	1.22 ± 0.29	1.342 (0.26)
TG, mmol/L	2.14 ± 0.91	2.03 ± 0.84	2.18 ± 1.02	2.23 ± 0.93	3.099 (0.03)
LDL-C, mmol/L	2.90 ± 0.82	2.91 ± 0.84	3.00 ± 0.86	2.96 ± 0.86	1.592 (0.19)
Systolic BP, mmHg	119.06 ± 10.54	120.71 ± 10.19	119.61 ± 10.36	120.65 ± 10.29	0.487 (0.69)
Diastolic BP, mmHg	75.10 ± 6.48	77.00 ± 6.47	76.10 ± 6.31	76.15 ± 6.39	0.648 (0.58)
Variables	MDD with overweight (*N* = 1026)				
	1 (*N* = 270)	2 (*N* = 248)	3 (*N* = 253)	4 (*N* = 255)	X^2^ (*p*)
Gender					3.23 (0.36)
male, n (%)	89 (32.96%)	82 (33.07%)	80 (31.63%)	97 (38.04%)	
female, n (%)	181 (67.04%)	166 (66.93%)	173 (68.37%)	158 (61.96%)	
Education					10.62 (0.30)
Junior high school, n (%)	70 (25.93%)	74 (29.84%)	62 (24.51%)	52 (20.39%)	
High school, n (%)	118 (43.7%)	110 (44.36%)	106 (41.89%)	118 (46.27%)	
University degree, n (%)	69 (25.56%)	53 (21.38%)	69 (27.27%)	75 (29.41%)	
Master’s degree, n (%)	13 (4.81%)	11 (4.42%)	16 (6.33%)	10 (3.93%)	
Marital status					4.01 (0.26)
Single, n (%)	63 (23.33%)	61 (24.60%)	74 (29.25%)	78 (30.59%)	
Married, n (%)	207 (76.67%)	187 (75.40%)	179 (70.75%)	177 (69.41%)	
Suicide attempts					5.45 (0.14)
No, n (%)	227 (84.07%)	195 (78.63%)	205 (81.03%)	198 (77.65%)	
Yes, n (%)	43 (15.93%)	53 (21.37%)	48 (18.97%)	57 (22.35%)	
Anxiety symptoms					3.67 (0.30)
No, n (%)	237 (87.78%)	213 (85.89%)	222 (87.75%)	215 (84.31%)	
Yes, n (%)	33 (12.23%)	35 (14.11%)	31 (12.25%)	40 (15.69%)	
Psychological symptoms					4.10 (0.25)
No, n (%)	247 (91.49%)	221 (89.11%)	223 (88.14%)	225 (88.24%)	
Yes, n (%)	23 (8.52%)	27 (10.89%)	30 (11.86%)	30 (11.76%)	
TSH abnormality					7.12 (0.07)
No, n (%)	91 (33.70%)	82 (33.07%)	86 (33.99%)	66 (25.88%)	
Yes, n (%)	179 (66.30%)	166 (66.94%)	167 (66.01%)	189 (74.12%)	

1:BMI≤24.48; 2: 24.48<BMI≤25.26; 3: 25.26<BMI≤26.28; 4: 26.28<BMI.

**Table 3:** The multicollinearity analysis between variables.

	Non-standardised coefficient		Standard coefficient			Covariance statistic	
	B	SE		t	Sig.	tolerance	VIF
(Constants)	4.76	.53		8.96	.00		
Age	.01	.08	.17	.11	.91	.00	2937.92
Illness duration, months	-.01	.01	-.05	-.85	.39	.22	4.50
Age of onset, years	-.01	.08	-.11	-.07	.94	.00	2885.29
Gender	-.03	.04	-.02	-.65	.52	.96	1.04
Education	.00	.02	.00	.13	.90	.77	1.30
Marital status	-.05	.06	-.04	-.95	.34	.53	1.89
HAMD	.00	.01	.01	.11	.92	.32	3.18
HAMA	-.01	.00	-.06	-1.15	.25	.32	3.10
Psychotic positive score	-.02	.02	-.17	-1.58	.11	.07	15.21
TSH, μIU/L	.09	.02	.35	6.34	.00	.25	4.05
TgAb, IU/L	.06	.07	.04	.86	.39	.35	2.89
TPOAb, IU/L	.00	.00	.06	1.63	.10	.71	1.42
FT3, pmol/L	-.01	.03	-.01	-.48	.63	.89	1.12
FT4, pmol/L	-.00	.01	-.02	-.67	.50	.91	1.11
TC, mmol/L	-.04	.03	-.06	-1.41	.16	.41	2.46
TG, mmol/L	.04	.02	.06	2.03	.04	.85	1.17
HDL-C, mmol/L	-.06	.07	-.03	-.89	.37	.81	1.24
LDL-C, mmol/L	.08	.03	.11	3.11	.00	.58	1.72
Systolic BP, mmHg	.00	.00	.06	1.19	.23	.27	3.65
Diastolic BP, mmHg	-.00	.00	-.01	-.13	.90	.46	2.18
BMI	-.010	.01	-.02	-.67	.50	.95	1.06

Dependent variable: Fasting glucose.

### The related factors for elevated fasting glucose in overweight/obese MDD patients

We explored related factors for elevated fasting glucose in overweight/obese patients with MDD. We checked for multicollinearity between variables, and the results are shown in **Table 3**. We included five variables (Age, TSH, TgAb, TPOAb, TG) in the multivariate logistic regression. As shown in **Table 4**, the following four factors for elevated fasting glucose in overweight/obese MDD patients were identified: age (B = -0.57, P = 0.02, OR = 0. 58), TSH (B = 0.37, P < 0.001, OR = 1.45),19 TgAb (B = -0.001. P = 0.02, OR = 1.00), TPOAb (B = 0.001, P = 0.01, OR = 1.00), and TG20 (B = 0.23, P = 0.02, OR = 1.26).

**Table 4:** Factors associated with elevated fasting glucose in overweight/obese MDD patients.

Variables	B	Wald statistic	p value	OR	95%CI
Age	-0.57	5.96	0.02	0.58	0.36-0.90
TSH, μIU/L	0.37	71.58	<0.001	1.45	1.33-1.58
TgAb, IU/L	-0.001	5.58	0.02	0.999	0.99-1.00
TPOAb, IU/L	0.001	6.13	0.01	1.00	1.00-1.00
TG, mmol/L	0.23	5.60	0.02	1.26	1.04-1.51

TSH, thyroid stimulating hormone; TgAb, antithyroglobulin; TPOAb, thyroid peroxidases antibody; TG, triglycerides.

Multivariate logistic regression was used to analyze

**Figure 2:**
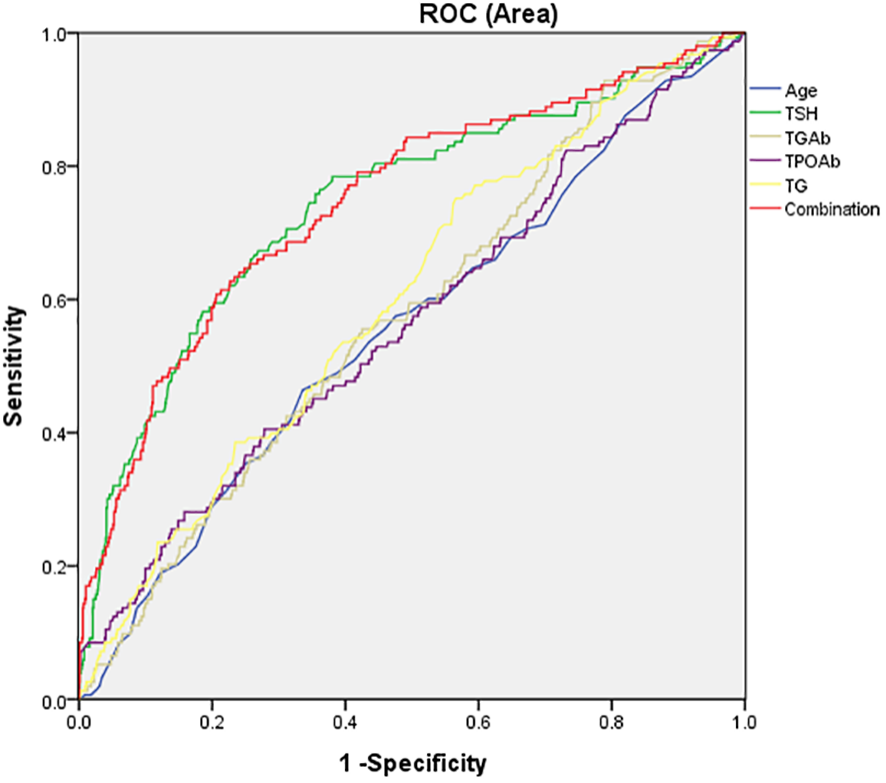
The potential capacity of related factors for discriminatory between overweight/obese MDD patients with elevated fasting glucose and normal fasting glucose. The area under the curve of TSH, TG, TgAb, TPOAb, and combination were 0.743, 0.601, 0.576, 0.566, and 0,747, respectively.

Next, AUCROC was used to assess whether the variables in **Table 4** could distinguish between patients with elevated fasting glucose and those with normal fasting glucose. As shown in **Figure 2**, the area under the curve of TSH, TG, TgAb, TPOAb, were 0.743, 0.601, 0.576, and 0.566, respectively. The AUC value of combination all these four parameters was 0.747. The results indicated that the AUC value of TSH and combination was highest, which could distinguish patients with elevated fasting glucose from those with normal fasting glucose.

### Correlation of elevated fasting glucose with clinical and metabolic parameters in overweight/obese MDD patients

The results of multifactorial regression analysis showed that TSH (B=0.10, t=6.38, *P*<0.001), TG (B=0.04, t=1.10, *P*=0.05), and LDL-C (B=0.08, t=3.08, *P*=0.02) were independently associated with elevated fasting glucose levels.

## Discussion

To our knowledge, this is the first cross-sectional study with a large sample size to explore the prevalence and clinical correlates of elevated fasting glucose in overweight/obese FEDN MDD patients. The main findings of this study were: (1) elevated fasting glucose in overweight/obese MDD patients was associated with TSH, TgAb, TPOAb, and TG; (2) TSH had the potential to differentiate the MDD patients with elevated fasting glucose from those with normal fasting glucose; and (3) TSH, TG, and LDL-C were independently associated with elevated fasting glucose in overweight/obese MDD patients.

Our study showed that TSH, TG and LDL-C were factors associated with elevated fasting glucose in overweight/obese FEDN MDD patients, with the elevated fasting glucose group being significantly higher than the normoglycemic group. The reasons and mechanisms for this phenomenon may be as follows: First, the concentration of 5-hydroxytryptamine in the brain is reduced in patients with major depression, leading to an increase in the concentration of thyrotropin-releasing hormone (TRH), which accelerates the secretion of TSH (28). Increased serum TSH can then act on TSH receptors in adipocytes to inhibit adipose triglyceride lipase, which in turn causes increased storage of triglycerides and an increase in adipocyte size, ultimately leading to overweight or obesity (29). Excess adipose tissue, especially large abdominal accumulations of dysfunctional fat, in turn increases triglyceride and LDL-C production, ultimately triggering insulin resistance and glucose dysregulation (30, 31); thus, elevated TSH, TG, and LDL-C may trigger elevated fasting glucose in overweight/obese FEDN MDD patients. However, some details are unknown and more large sample investigations and animal experiments are needed to prove this.

Our study found that overweight/obese MDD patients were more likely to have elevated fasting glucose levels compared to patients with normal BMI, indicating an increased risk of developing diabetes, and the results of some previous studies are consistent with ours. For example, Haleem et al. reported that obese MDD patients had higher fasting glucose levels than non-depressed obese patients and that depression severity was positively associated with elevated serum glucose (18). Zhang et al. found that overweight MDD patients had higher fasting glucose levels than non-overweight MDD patients at different ages of onset (32). Our results also showed that MDD patients with elevated fasting glucose also had significantly higher TSH, TPOAb, TC, TG, LDL-C, and systolic and diastolic blood pressure compared to patients with normal fasting glucose. These differences suggest that the overall severity of metabolic disorders is also increased in MDD patients with abnormal fasting glucose.

The association of depressive symptoms with dysregulated glucose metabolism in never-treated MDD patients involves an imbalance in the neuro-endocrine network and, as a result, increasing attention has been paid to neuroendocrine mechanisms. First, one of the main triggers of glucose metabolism disorders due to overweight/obesity has been reported to be insulin resistance. Patients with MDD are at particularly high risk of developing overweight or obesity (33), and abdominal fat accumulation induces insulin resistance, meaning that overweight or obese MDD patients are more likely to develop glucose metabolism abnormalities (34). Furthermore, the reduction of depressive symptoms was positively correlated with the remission of insulin resistance (35). The insulin sensitizer pioglitazone alone significantly improved depressive symptoms in MDD patients with abdominal obesity after 12 weeks of treatment (35). Insulin also produces antidepressant effects by reducing visceral fat (36). However, it has also been reported that there is little association between insulin resistance and depression (37–39). Second, one of the endocrine signs of MDD is an abnormally increased activity of the hypothalamic-pituitary-adrenal system (HPA axis), followed by cortisolism. Cortisol is a well-known anti-insulin hormone. Sustained high levels of cortisol increase the intake of high-calorie foods and drive the accumulation of visceral fat. The endocrine sequelae of hypercortisolism are mainly hyperglycemia and hyperinsulinemia (40). Third, elevated glucocorticoid concentrations, also a common endocrine marker in MDD, are closely associated with dyslipidemia, impaired glucose homeostasis, hypertension, and abdominal obesity (41). Taken together, multiple risk factors such as insulin resistance, hypercortisolism, and elevated glucocorticoid concentrations may be mechanisms underlying abnormal glucose metabolism in overweight/obese MDD patients, but the exact mechanisms are inconclusive and require subsequent in-depth studies.

In addition, this study showed that higher HAMA score was positively associated with elevated fasting glucose in overweight/obese MDD patients. Several studies have reported a relationship between anxiety symptoms and glucose metabolism, but the results are not entirely consistent. For example, Simon et al. found a causal relationship between anxiety symptoms and weight gain or obesity (42), which in turn was associated with elevated glucose and insulin concentrations (43, 44). Abnormally elevated glucose can further interfere with an individual’s mood (45). However, Jaremka et al. reported no change in glucose levels in individuals with significant anxiety symptoms in those with larger waist circumference (46).

The present study has some limitations. First, the present study used a cross-sectional design, so the results did not explain the causal relationship between elevated fasting glucose and BMI in patients with MDD. Future studies should focus on longitudinal studies with the aim of further exploring the relationship between BMI and glucose metabolism in MDD. Second, studies have shown that fasting glucose alone is not sufficient to diagnose abnormal glucose metabolism. Moreover, HbA1c and oral glucose tolerance test (OGTT) are more sensitive measures of abnormal glucose metabolism than fasting glucose (47). However, unfortunately, in the present study, we did not collect data on HbA1c and OGTT and therefore could not fully elucidate further conclusions on impaired glucose metabolism in MDD patients. Third, unhealthy lifestyles, especially high-calorie diet and sedentary lifestyles, may make patients more susceptible to metabolic disorders such as hyperlipidemia, hypertension, and metabolic syndrome (48, 49). Unfortunately, we did not collect data in this area, which should be included in future studies. Fourth, a healthy control group was not set up in this study. In addition, the prevalence of metabolic disorders was higher among the immediate family members of MDD patients compared to healthy controls, and unfortunately (50), we did not investigate the family history of diabetes in the participants in this study, which should be corrected in future studies. Fifth, the sample included in this study consisted of patients with FEDN MDD, so the conclusions drawn cannot be extended to MDD patients with a history of recurrent episodes and medication use.

Identification of accurate risk factors is a promising approach to effectively prevent elevated fasting glucose in overweight/obese MDD patients. Our findings show that the prevalence of elevated fasting glucose is much higher in overweight/obese FEDN MDD patients than in MDD patients with normal BMI. Age, disease duration, TSH, TgAb, TPOAb and TG were related factors for elevated fasting glucose in overweight/obese FEDN MDD patients. These results are important for clinical practice because changes in metabolic markers might be a biomarker of elevated fasting glucose. Second, the prevalence of elevated fasting glucose was higher in overweight/obese FEDN MDD patients with high TSH, TG, and LDL-C levels.

In conclusion, our results showed that the prevalence of elevated fasting glucose in overweight/obese FEDN MDD patients was much higher than that in MDD patients with normal BMI. Second, TSH, TgAb, TPOAb, and TG are related factors for elevated blood glucose in overweight/obese FEDN MDD patients.

## Data availability statement

The original contributions presented in the study are included in the article/supplementary material. Further inquiries can be directed to the corresponding authors.

## Ethics statement

The studies involving human participants were reviewed and approved by the ethics committee of the First Hospital of Shanxi Medical University. The patients/participants provided their written informed consent to participate in this study.

## Author contributions

WG wrote the original manuscript. ZD, XC, and DZ analyzed the data. HX and XZ supervised the project and revised the manuscript. All authors contributed to the article and approved the submitted version.
